# Phenotypic and genomic analysis of antimicrobial resistant *Escherichia coli* isolates obtained from starter-phase broilers of commercial chicken farms in central Ethiopia

**DOI:** 10.3389/fmicb.2026.1848618

**Published:** 2026-06-19

**Authors:** Abebe Wirtu, Gunther Antonissen, Hika Waktole, Bizunesh Mideksa, Amanda Hettiarachchi, Sieglinde Coppens, Sebastiaan Theuns, Filip Boyen

**Affiliations:** 1 Department of Pathobiology, Pharmacology and Zoological Medicine, Faculty of Veterinary Medicine, Ghent University, Merelbeke-Melle, Belgium; 2 Department of Clinical Studies, College of Veterinary Medicine and Agriculture, Addis Ababa University, Bishoftu, Ethiopia; 3 Department of Microbiology, Parasitology, and Poultry Health, College of Veterinary Medicine and Agriculture, Addis Ababa University, Bishoftu, Ethiopia; 4 Department of Veterinary Science, School of Veterinary Medicine, Ambo University, Ambo, Ethiopia; 5 PathoSense BV, Ghent, Belgium

**Keywords:** antimicrobial resistance, broiler chickens, commercial farms, *Escherichia Coli*, Ethiopia, virulence gene

## Abstract

Antimicrobials are widely used in intensive poultry production, particularly in sub-Saharan Africa, promoting the selection and spread of antimicrobial-resistant bacteria. Avian fecal *Escherichia coli* (AFEC) act as important reservoirs of antimicrobial resistance (AMR) genes, reflecting baseline resistance levels and facilitating dissemination of resistance and virulence determinants within poultry production systems. This study aimed to phenotypically and genotypically characterize cloacal *E. coli* recovered from apparently healthy broiler chickens in Ethiopia, focusing on AMR profiles and virulence-associated genes. A total of 485 cloacal swabs were collected from 52 commercial broiler farms after brooding (21 ± 1 days of age), with 9–11 swabs per farm. Antimicrobial susceptibility testing was performed on 163 *E. coli* isolates using the Sensititre EUVSEC3 microdilution plate against 15 antimicrobials. Whole-genome sequencing (WGS) was conducted on a subset of 20 isolates representing different resistance profiles. Overall, 256 swabs (53%) yielded *E. coli*, confirmed by MALDI-TOF MS. Using clinical breakpoints, resistance was most frequent to tetracycline (84%), nalidixic acid (74%), ciprofloxacin (72%), and trimethoprim (53%). No resistance was detected to ceftazidime, cefotaxime, colistin, or meropenem. Multidrug resistance (MDR) was present in (77%) of isolates, indicating a substantial resistance burden. Based on epidemiological cut-off (ECOFF) values, acquired resistance was highest for ciprofloxacin (96%), followed by tetracycline (84%), nalidixic acid (77%), and trimethoprim (54%), while resistance to aminoglycosides, azithromycin, and tigecycline remained low (<10%). WGS identified multiple AMR determinants consistent with phenotypic resistance, including frequent mutations in gyrA, parC, and parE associated with fluoroquinolone resistance. Isolates displayed considerable genetic diversity with small clusters of closely related strains. Plasmid profiling revealed recurrent IncFIB, IncFII, IncI1-I, and IncX replicon types, with most isolates carrying at least one conjugative plasmid encoding AMR genes or APEC-associated virulence determinants, particularly on IncF and IncX plasmids. Overall, Ethiopian broiler farms harbor diverse *E. coli* populations with high AMR prevalence and mobile genetic elements, highlighting the need for strengthened antimicrobial stewardship, improved biosecurity, enhanced diagnostics, and integrated AMU–AMR surveillance in the poultry sector.

## 1 Introduction

Antimicrobials are extensively used in poultry production worldwide to treat diseases, improve productivity, and unfortunately also for disease prevention and growth promotion (Ibrahim et al., 2023a; Van Boeckel et al., 2015). In sub-Saharan Africa, multiple classes of antimicrobials are widely and frequently used in intensive poultry production farms (Azabo et al., 2022). However, mounting evidence shows that heavy reliance on antimicrobials leads to the selection, emergence, and dissemination of resistant bacterial strains (Ye et al., 2025). This issue is particularly evident in poultry production, where antibiotic use often promotes the development of resistant bacteria, with young birds being especially vulnerable (Diarra and Malouin, 2014). Frequent antimicrobial use during this early rearing phase has been widely reported and is closely linked to the emergence and dissemination of resistance, undermining poultry productivity and posing risks to public health (Abreu et al., 2023; Tiseo et al., 2020; Van Boeckel et al., 2015). Such resistance emerges when bacteria acquire the ability to withstand antimicrobial agents through mutation or horizontal gene transfer (von Wintersdorff et al., 2016; Wang, 2023). More broadly, the World Health Organization (WHO) reports that antimicrobial resistance (AMR) in major bacterial pathogens has reached alarming levels worldwide. For example, resistance in *Escherichia coli* and *Klebsiella* spp. to last-resort antibiotics including third-generation cephalosporins and carbapenems has reached up to 54%, while surveillance capacity remains limited in many low and middle-income countries, such as those in sub-Saharan Africa (Iwu-Jaja et al., 2021; World Health Organization [WHO], 2022). Economic projections further warn that AMR related losses may escalate dramatically, potentially reaching trillion-dollar levels by the mid-21st century (Ahmed et al., 2018). In recognition of its wide ranging implications for global health, food system and socio-economic stability, combating AMR is also explicitly incorporated into the United Nations’ 2030 Agenda for Sustainable Development (Tang et al., 2017).

Avian pathogenic *Escherichia coli* (APEC) is a major cause of disease in broiler production systems worldwide, including in Africa (Lúcio et al., 2025; Mohamed et al., 2018). Although research in Ethiopia remains limited, existing studies indicate that APEC strains exhibit genomic plasticity that facilitates the acquisition of AMR through mutation and horizontal gene transfer (Messele et al., 2017). Consequently, APEC contributes to the circulation of multidrug-resistant *E. coli* within poultry operations and to resistance against commonly used antibiotics (Apostolakos et al., 2021; Kathayat et al., 2021; Mehat et al., 2021). However, APEC strains are not the sole drivers of resistance in poultry. Avian fecal *Escherichia coli* (AFEC) represent the dominant *E. coli* population in the intestinal tract of healthy birds and serve as a major reservoir of AMR genes. Because these mainly commensal strains form a large proportion of the gut microbiota and are continuously exposed to antimicrobial selection pressures, they readily accumulate and disseminate resistance determinants (Rezatofighi et al., 2021; Starčič Erjavec et al., 2017). Importantly, AFEC strains not only harbor diverse AMR genes but are also widely utilized as standard indicator organisms for AMR surveillance in poultry systems, offering valuable insight into the baseline resistance prevalence within flocks and production environments (Bhattarai et al., 2024; Mulchandani et al., 2024). Both APEC and AFEC populations therefore contribute to the dissemination of resistance within poultry production. While APEC-associated AMR directly affects clinical disease management, AFEC strains primarily facilitate the spread of resistance genes and virulence factors within poultry flocks and into human exposure pathways at the animal-human interface, representing an important One Health concern (Hu et al., 2025; Patel et al., 2024; Rezatofighi et al., 2021). In Ethiopia, commercial broiler industry is expanding rapidly to meet nutrition demand, support income generation, and create employment opportunities (Sime, 2022; Tirfie, 2021). Despite this intensification, farm management practices remain generally suboptimal, characterized by weak biosecurity practices and limited diagnostic capacities which hinder effective disease prevention and control, thereby allowing resistance to persist and intensify (Asfaw et al., 2021; Tadesse et al., 2024; Waktole et al., 2023). Although Ethiopia has established regulatory frameworks governing veterinary antimicrobial use including manuals for pharmaceutical management, enforcement of rules related to veterinary drug distribution and prescription remains inadequate. As a result, antimicrobials are often sold without veterinary oversight, further exacerbating the risk of inappropriate use and AMR development (Etefa et al., 2021; Gemeda et al., 2020; Beyene et al., 2016).

Despite these concerns, the dynamics of AMR within Ethiopia’s commercial broiler sector remain largely unexplored. Only a limited number of studies have reported the occurrence of AMR in Ethiopian poultry production systems (Akalu et al., 2024; Bushen et al., 2021; Shecho et al., 2017; Tigabie et al., 2023; Waktole et al., 2024). Although these studies provide valuable insights, most were conducted in a small number of farms or backyard production systems and predominantly focused on older birds. Furthermore, virulence profiles and genomic mechanisms underlying AMR were often not investigated, limiting a deeper understanding of the relevance of resistant isolates for poultry production and public health. In response to the rising AMR concerns, the recently endorsed Multidrug Organisms Prevention and Containment Guideline (Ministry of Agriculture [MOA], 2026) for the Agri-food Sector emphasizes the need to reduce the emergence and spread of multidrug-resistant organisms (MDROs) in Ethiopia through integrated prevention and containment activities implemented in farms, abattoirs, aquaculture, food of animal and plant origin, and animal healthcare facilities. Nevertheless, evidence of AMR occurrence in the poultry industry, particularly in commercial broiler farms remains limited (Tilahun and Efa, 2026). This limitation also highlights the need for more detailed investigation of the early production stages. Therefore, the objective of this study was to phenotypically and genotypically characterize avian *E. coli* (AFEC) isolated from apparently healthy broiler chickens during starter-phase in Ethiopia with a focus on AMR and virulence-associated genes.

## 2 Materials and methods

### 2.1 Study area

**FIGURE 1:**
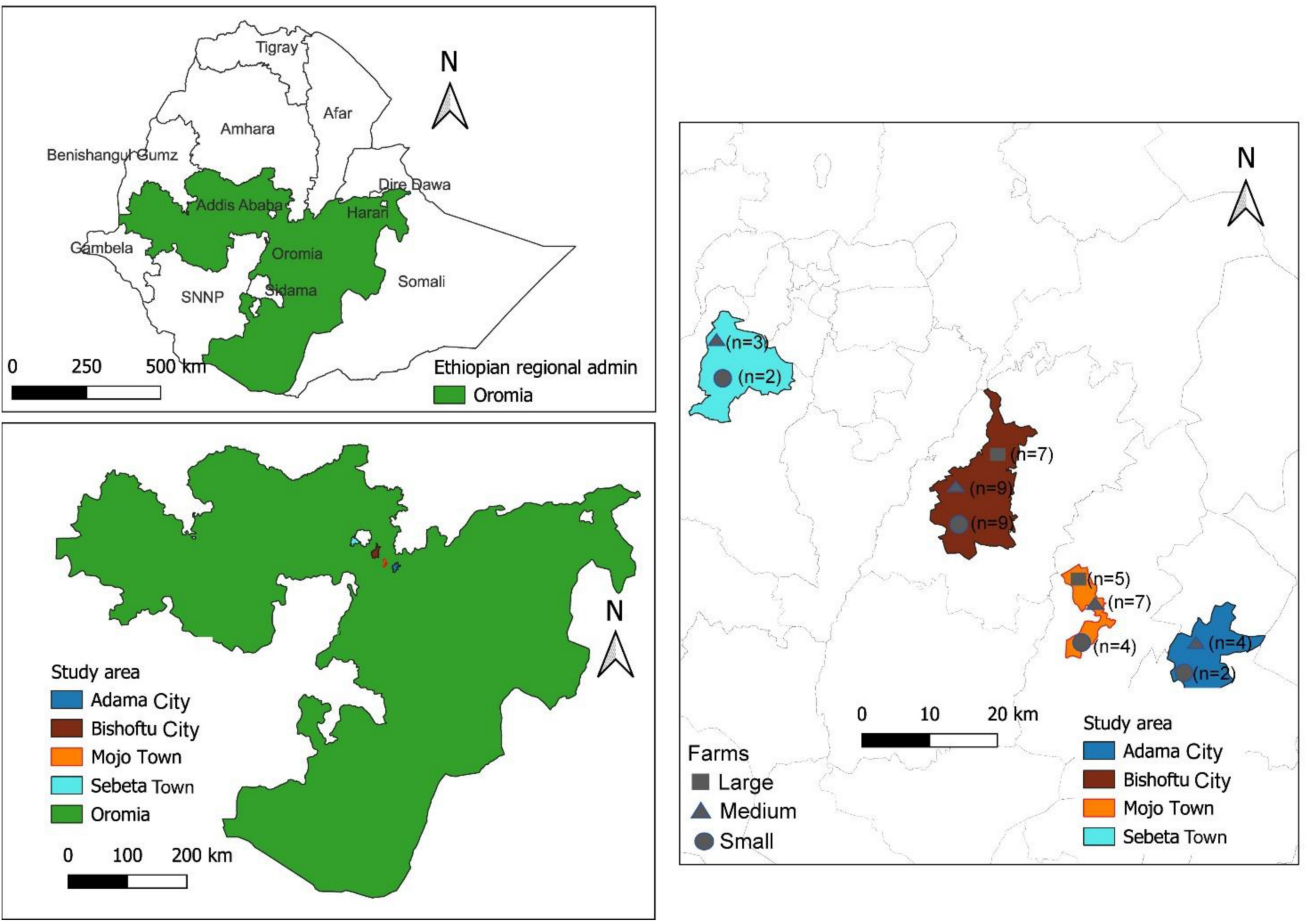
Map of Ethiopia and Oromia Regional State depicting the locations of study areas.

A non-probability convenience sampling study was carried out in Central Ethiopia, namely in the areas of Bishoftu, Adama, Mojo, and Sebata (Figure 1). These urban areas were selected based on the presence of a large number of commercial broiler farms and on their proximity to the College of Veterinary Medicine and Agriculture of the Addis Ababa University, where bacteriological isolation was done. The areas are characterized by a subtropical mid to highland climate, elevations ranging from approximately 1,700–2,400 meters above sea level, and a bimodal rainfall pattern (Ethiopian Meteorological Institute [EMI], 2024). The commercial intensive and semi-intensive broiler and layer operations of the country are concentrated in these areas due to their proximity to market centers, access to major transportation routes, and availability of veterinary and hatchery services.

### 2.2 Study design and study farms

A cross-sectional study was carried out on broiler farms located in the previously described districts-urban areas of Central Ethiopia from March 2024 to January 2025. The study included commercial broiler farms operating varying scales in the study areas. Broiler flocks were sampled after the brooding period (chicks were 21 ± 1 days old), which corresponds to the starter phase in local management practices. Commercial broiler farms with flock sizes greater than 1,000 birds, registered and actively operating in the study areas were identified using farm lists obtained from the respective Urban Bureaus of Agriculture. According to Tadesse et al. (2024) farms were stratified by production scale as follows: (i) Large-scale farms: more than 10,000 birds; (ii) Medium-scale farms: 5,000–10,000 birds; and (iii) Small-scale farms:1,000–5,000 birds per batch.

Before sampling, a preliminary survey was conducted on approximately 10% of poultry farms in the study area to assess management practices and owner’s awareness of poultry husbandry and biosecurity (unpublished data). The willingness of farm owners to participate in the study was enquired. Informed consent was obtained from all selected farms prior to sample collection. In total, 52 broiler farms were included in the study: 12 large scale, 23 medium scale, and 17 small-scale farms (Figure 1).

### 2.3 Sample collections and isolation of *E. coli*

Cloacal swab samples were collected aseptically from apparently healthy chickens. Approximately 10 cloacal swabs were obtained per farm with minor (±1) variation depending on the ease of handling individual birds during sampling. Sampling was performed using commercially prepared Meus^®^ swab kits (MEUS S.r.l., Italy), consisting of sterile cotton swabs in polypropylene tubes prefilled with Amies transport medium (AMIES CLR). After collection, each swab was placed into its corresponding tube, individually labeled and transported in ice-pack cooled boxes to the Microbiology Laboratory of College of Veterinary Medicine and Agriculture of the Addis Ababa University, Ethiopia.

Isolation of *E. coli* from the cloacal swabs was carried out following conventional bacteriological procedures described by Clinical and Laboratory Standards Institute [CLSI] (2008). The samples were inoculated onto MacConkey agar (No. 3, Oxoid) and incubated at 37 °C for 24 h. Lactose positive colonies with a pink to red color were purified on MacConkey agar (Oxoid), and subsequently streaked onto Eosin Methylene Blue (EMB) agar and incubated at 37 °C for 24 h. Presumptive *E. coli* colonies appearing as dark violet with black centers and a greenish metallic shine were transferred to Brain Heart Infusion (BHI) broth, containing 30% sterile glycerol, in properly labeled 1.8 mL cryovial tubes and stored at −20 °C in a cryovial box. The preserved samples were shipped to the Bacteriology Laboratory, Faculty of Veterinary Medicine, Ghent University, Belgium, for further identification, antimicrobial susceptibility testing and whole genome sequencing.

### 2.4 Identification of *E. coli* and antimicrobial susceptibility testing

At the laboratory of Bacteriology, Faculty of Veterinary Medicine, Ghent University, Belgium, the bacterial isolates tentatively identified as *E. coli* were purified on Columbia agar supplemented with 5% sheep blood and species identification was done using MALDI-TOF mass spectrometry as described previously (Vera et al., 2019).

Antimicrobial susceptibility of the confirmed *E. coli* isolates was conducted using a Sensititre EU Surveillance *E. coli* EUVSEC3 Plate (Trek Diagnostic Systems, Thermofisher Scientific, Merelbeke, Belgium), following the manufacturer’s instructions. Briefly, 1–3 colonies were suspended in sterile saline and adjusted to match a 0.5 McFarland standard. From this suspension, 10 μL was inoculated into 11 mL sterile Cation Adjusted Mueller Hinton broth (Thermo Scientific Sensititre, Merelbeke, Belgium). After vortexing, 50 μL of the Mueller-Hinton broth with bacteria was dispensed into each well of the Sensititre micro-titer plate pre-loaded with the lyophilized antimicrobials using a multichannel pipette (final concentration of 2.5 × 10^4^ CFU/well). The microplate was incubated at 35 °C for 18 h, after which growth end-points were established for each antimicrobial to provide the minimum inhibitory concentrations (MIC). The MIC was defined as the lowest antimicrobial concentration at which no visible growth could be observed. Quality control (QC) was maintained using the reference strains *E. coli* ATCC 25922 and *S. aureus* ATCC 29213. MIC testing was conducted against 15 antimicrobials (Table 1) on 163 *E. coli* isolates, corresponding to 3–4 isolates through systematic random selection from each farm. Interpretation of MIC values was based on (1) the epidemiological cut-off values (ECOFFs) set by the European Committee on Antimicrobial Susceptibility Testing (Eucast Clinical Breakpoints and Dosing of Antibiotics [EUCAST], 2025) and (2) human clinical breakpoints (Clinical and Laboratory Standards Institute [CLSI], 2025), as poultry-specific breakpoints were unavailable for most antimicrobial agents (Table 1).

**TABLE 1:** Panel of antimicrobials and concentration ranges included in antimicrobial susceptibility testing and applied epidemiological cut-offs (ECOFFs) and human clinical breakpoints.

Antimicrobial agent (abbreviation)	Concentration range tested (mg/L)	Non-wild type population* (mg/L)	Clinical breakpoint for susceptibility^#^ (mg/L)	Clinical breakpoint for resistance^#^ (mg/L)
Gentamicin (GEN)	0.5–16	≥2	≤2	≥16
Tetracycline (TET)	2–32	≥16	≤4	≥16
Ceftazidime (TAZ)	0.25–8	≥2	≤4	≥16
Trimethoprim (TMP)	0.25–16	≥4	≤8	≥16
Ampicilline (AMP)	1–32	≥16	≤8	≥32
Amikacin (AMI)	4–128	≥16	≤4	≥16
Chloramphenicol (CHL)	8–64	≥32	≤8	≥32
Azithromycin (AZI)	2–64	>32	NA	≥32
Nalidixic acid (NAL)	4–64	≥16	≤16	≥32
Colistin (COL)	1–16	≥4	NA	≥4
Cefotaxime (FOT)	0.25–4	≥0.5	≤1	≥4
Meropenem (MERO)	0.03–16	≥0.125	≤1	≥4
Ciprofloxacin (CIP)	0.015–8	≥0. 125	≤0.25	≥1
Tigecycline (TGC)	0.25–8	≥1	NA	NA
Sulfamethoxazole (SMX)	8–512	NA	≤256	≥512

NA indicates that information is not available.

*Indicates ECOFF (Epidemiological cut-off)^1^.

**#**Indicates clinical breakpoints (https://clsi.org/shop/standards/m100/).

According to the EUCAST database (most recently assessed on 02.06.2025^1^), isolates with MIC values equal to or above the ECOFF were classified as non-wild type, indicating the acquisition of resistance mechanisms, or in other words, acquired resistance. Isolates with MIC values between the clinical susceptibility and resistance breakpoints were considered intermediately susceptible, whereas those with MIC values exceeding the clinical resistance breakpoint were considered resistant, according to CLSI M100-ED35 guidelines (Clinical and Laboratory Standards Institute [CLSI], 2025). Due to the absence of an epidemiological cut-off value (ECOFF) for sulfamethoxazole, non-wild type was assumed based on the presence of bi- or multimodal MIC distribution patterns for this antimicrobial agent (Kahlmeter and Turnidge, 2022), where isolates with MIC values of 512 mg/L were interpreted as non-wild type, suggesting acquired resistance mechanisms. The results of the antimicrobial susceptibility tests were primarily interpreted using the EUCAST epidemiological cut-off values, which define whether an isolate has acquired resistance against a certain antimicrobial by distinguishing from the wild type population and are therefore the preferred phenotypic interpretation criterion to evaluate genotypic results (Ibrahim et al., 2023b). On the other hand, using human clinical breakpoints provides more precise insights into whether the observed susceptibility results are clinically relevant in human medicine. We therefore chose to report both interpretations.

The European Food Safety Authority (EFSA) defines AMR prevalence as the proportion of tested isolates of a given microorganism that are resistant to a given antimicrobial agent (European Food Safety Authority [EFSA], 2025). These levels are categorized as: rare (<0.1%), very low (0.1%–1%), low (>1%–10%), moderate (>10%–20%), high (>20%–50%), very high (>50%–70%), and extremely high (>70%). An isolate was considered as multi-drug resistant (MDR) when resistance was observed against at least one antimicrobial agent from at least three different antimicrobial classes.

### 2.5 Whole genome sequencing (WGS)

Out of the 163 isolates used for susceptibility testing, twenty *E. coli* isolates were selected to represent diverse AMR profiles, in order to maximize the information gained from the genomic analysis and prepared for WGS. Genomic DNA was extracted using the Quick-DNA Fungal/Bacterial Miniprep Kit (Zymo Research, United States) and prepared for long-read sequencing on Oxford Nanopore Technologies’ (ONT) PromethION device at PathoSense BV. Library preparation was performed using the SQK-RBK114-96 kit (ONT, United Kingdom) and sequenced on R10.4.1 flow cells (ONT, United Kingdom). Sequencing throughput and metrics are provided in Supplementary Table 1. Raw reads were filtered for a minimum length of 1,000 nucleotides minimum quality score of 10. Filtered reads were subjected to *de novo* bacterial genome assembly using Flye v2.9.3 (Kolmogorov et al., 2019) and assemblies were polished using medaka v1.12.0. Genome quality and completeness of final assemblies were evaluated with CheckM v1.2.4 (Parks et al., 2015) using the *Escherichia* specific marker gene database.

### 2.6 *In silico* analysis

Amrprofiler (Skoulakis et al., 2025) was used for the identification of AMR genes and resistance -associated mutations in core genes and rRNA genes. AMR genes were screened against the CARD, Resfinder, and the Reference Gene Catalog databases using a 60% threshold for identity and coverage, and a protein start position of 200. Point mutation screening was performed by comparison against the genome of *E. coli* K-12 substr. MG1655. For point mutation screening in core genes, the protein start position was set to 200 and distance from mutation to 5. rRNA mutation screening was performed with default parameters, where the distance from mutation parameter was set to 5.

Multi locus sequence typing (MLST) was performed using the CGE MLST webtool for *E. coli* #1 using default settings (Larsen et al., 2012)^2^ while serotype and cgMLST were predicted using the CGE SeroTypeFinder and cgMLSTFinder 1.2 for *E. coli* (Enterobase) tools, respectively using default settings (Banerjee et al., 2021; Joensen et al., 2015). The presence of virulence associated genes was predicted using the CGE VirulenceFinder 2.0 tool for *E. coli* using default settings (Malberg Tetzschner et al., 2020). Detection of plasmids was conducted using the CGE PlasmidFinder tool for Enterobacterales using default settings (Carattoli et al., 2014) and mlplasmids^3^ accessed on 9/23/2025. Plasmid mobility was predicted using MOB-suite v3.1.9. via the Solu platform (Robertson and Nash, 2018; Saratto et al., 2025).

Phylogenetic relationship among isolates was performed using CSI phylogeny 1.4 pipeline (Kaas et al., 2014) on the CGE platform using *E. coli* C600 as the reference genome. Resulting single nucleotide polymorphism (SNP) matrices were used to construct a maximum likelihood phylogenetic tree which was visualized and annotated using the Interactive Tree of Life (iTOL) v4 tool (Letunic and Bork, 2019). The APEC virulence level was estimated by quantifying the presence of a selection of 10 APEC-associated virulence genes (*iss, tsh, iroN, ompT, iutA, cvaC, hlyF, iucD, papG* (II/III), and *papC*) which are described to be more frequently reported in APEC isolates compared with non-pathogenic *E. coli*, based on an extensive review of the related literature (Ovi et al., 2023), where a higher number of virulence genes predicted a higher APEC virulence level.

### 2.7 Data management and analysis

Data collected during the study were entered into Microsoft Excel^®^ 2010 and double-checked for accuracy. Descriptive statistics were calculated in excel including frequencies, percentages and averages for categorical variables. The specific method employed is described in the text and/or in the relevant table or figure legend.

### 2.8 Ethical clearance

Institutional Review Board Statement: The study was reviewed and approved by The Institutional Animal Care and Use Committee of College of Veterinary Medicine and Agriculture of the Addis Ababa University, Ethiopia (Protocol Ref. No: VM/ERC/03/45/17/2025). Informed Consent was obtained from all farm owners or managers included in the study in line with the approved protocol. Access to and use of biological materials originating from Ethiopia complied with the Nagoya Protocol and relevant national legislation. Samples were exported for non-commercial academic research under institutional agreements between Addis Ababa University and Ghent University, Belgium, with all required authorizations obtained, including import approval from the Federal Agency for the Safety of the Food Chain (FAVV), Belgium (permit no. 2025DBP167).

## 3 Results

Out of 485 cloacal swab samples (large-scale = 123; medium-scale = 209; small-scale = 153), 256 (53%) yielded recoverable *E. coli* isolates under the applied culture conditions. All presumptive isolates were subsequently confirmed as *E. coli* by MALDI-TOF mass spectrometry. The number of confirmed *E. coli* isolates recovered per farm ranged from 3 to 6. In terms of farm category, the proportions of isolates were 48% from large-scale farms, 55% from medium-scale farms, and 55% from small-scale farms (Supplementary Table 2).

### 3.1 Antimicrobial susceptibility results

All MIC values of the QC strains were within the acceptable ranges described by Clinical and Laboratory Standards Institute [CLSI] (2025). Results of antimicrobial susceptibility tests (MIC distributions) are summarized in Table 2. Of a total of 163 isolates tested against 15 antimicrobials, 159 (98%) were non-wild type showing acquired resistance to at least one antimicrobial based on ECOFF values. Similarly, 152 (93%) of the isolates were resistant to at least one antimicrobial agent according to clinical breakpoints, reflecting potential therapeutic failure in those isolates. The results revealed that 125 (77%) of the *E. coli* isolates exhibited multi-drug resistance (MDR). The highest proportion of acquired resistance (ECOFF based) was observed against ciprofloxacin (96%), followed by tetracycline (84%), nalidixic acid (77%) and trimethoprim (54%), while low levels (<10%) of acquired resistance were recorded for aminoglycosides, azithromycin and tigecycline. In contrast, when interpreted according to clinical breakpoints, the highest proportion of resistance was detected against tetracycline (84%) followed by nalidixic acid (74%), ciprofloxacin (72%), and trimethoprim (53%), respectively. No isolates showed acquired resistance to ceftazidime, colistin, cefotaxime, or meropenem.

**TABLE 2:** Minimum inhibitory concentration distribution for *E. coli* Isolates in broiler chickens.

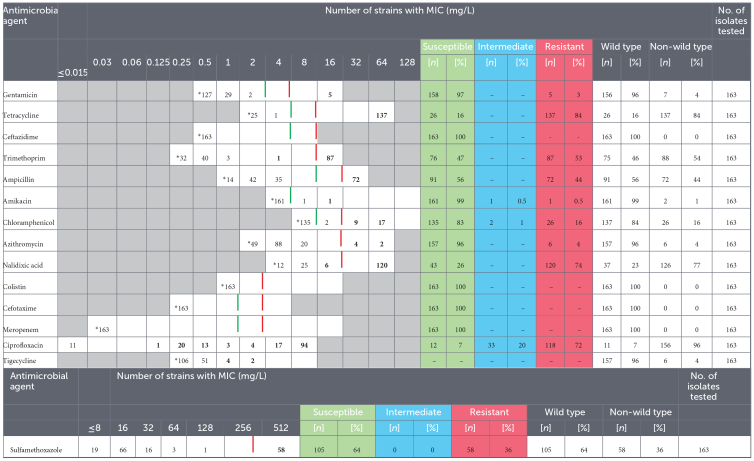

*No visible growth at this concentration means minimum inhibitory concentrations (MIC) is equal to or below this concentration. The Clinical and Laboratory Standards Institute (CLSI) breakpoint for susceptibility is depicted by a green vertical line, the CLSI breakpoint for resistance is depicted by a red vertical line. In case clinical breakpoints for susceptibility and resistance collide (nalidixic acid and trimethoprim) or only a clinical breakpoint for resistance is described (azithromycin, colistin), only a red line is shown. Non-wild type strains, either defined by epidemiological cut-offs (ECOFF) or by a bimodal distribution (sulfamethoxazole) are stated in bold. The colors represent different antimicrobial susceptibility categories, as defined by the applied interpretative criteria.

### 3.2 Whole genome sequencing (WGS) results

**FIGURE 2:**
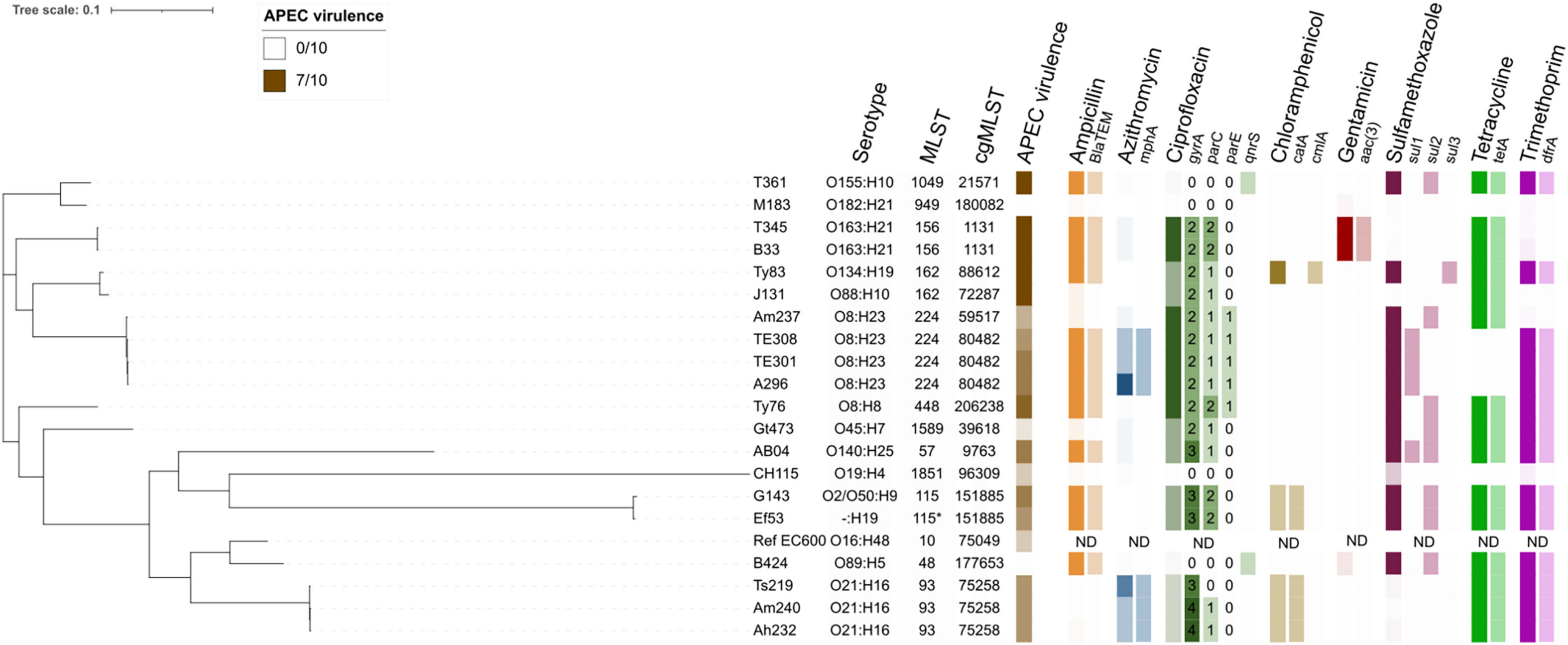
Illustrates the phylogenomic tree relationships among the 20 sequenced *E. coli* isolates, showing serotype, multi locus sequence typing (MLST), core genome multi locus sequence typing (cgMLST), and antimicrobial resistance (AMR) profiles of antimicrobial agents for which both phenotypic and genotypic results are available. The APEC associated virulence level was estimated by quantifying the presence of a selection of 10 virulence genes [*iss*, *tsh*, *iroN*, *ompT*, *iutA*, *cvaC, hlyF*, *iucD*, *papG* (II/III), and *papC*] which are described to be more frequently reported in APEC isolates compared with non-pathogenic *E. coli* (Ovi et al., 2023). The color shading in the antibiotic columns indicates minimum inhibitory concentrations (MIC) values as a gradient with white to pale shadings representing strains belonging to the wild type population and intensely colored shadings representing strains with highest MIC values, while the related AMR gene panel displays the presence (colored) or absence (white) of individual resistance genes. Mutations in *gyrA*, *parC*, and *parE* are shown numerically, also highlighting variation in the number of point mutations per gene as a color gradient.

Whole genome sequencing was performed on 20 *E. coli* isolates, selected from the 163 isolates used for susceptibility testing, representing different farms and AMR profiles, as shown in Table 3. Notable genetic heterogeneity was observed across the 20 sequenced *E. coli* isolates with between-isolate differences ranging between 4 and 48,462 SNPs (Figure 2 and Supplementary Table 3). Three focal clusters showed highly similar genetic profiles between isolates B33 and T345, isolates A296, TE301, TE308 and to a somewhat lesser extent Am237 and isolates Am240, Ah232, and Ts219. In the 20 sequenced isolates, fifteen different serotypes were observed with O21:H16 (four isolates), O8:H23 (three isolates) and O163:H21 (two isolates) most commonly detected (Figure 2). Similarly, MLST analysis identified several sequence types (ST) with ST224 (four isolates), ST93 (three isolates) and ST156 and ST162 (two isolates each) being most common (Figure 2). Isolates belonging to the same ST often displayed differences in plasmid profiles and resistance gene content (Tables 4, 5). Nevertheless, some isolates within these ST groups shared highly similar plasmid configurations and overlapping resistance or virulence gene patterns. Plasmid analysis revealed the frequent occurrence of IncFIB, IncFII, IncI1-I, and IncX plasmid replicon types. Sixteen out of 20 sequenced isolates carried at least 1 conjugative plasmid with AMR or APEC-associated virulence genes (Table 5). Two isolates did not carry any plasmid-mediated APEC-associated virulence genes or AMR genes, consistent with their phenotypic susceptibility, while two isolates carried only non-mobilizable plasmids containing APEC-associated virulence genes or AMR genes (Table 5).

**TABLE 3:** Overview of 20 selected *E. coli* isolates with their respective source farms.

Strain number	Farm of origin	Farm operation
A296	Al31	Medium scale
AB04	A1	Large scale
Ah232	Ah24	Medium scale
Am237	Am25	Medium scale
Am240	Am25	Medium scale
B33	B4	Small scale
B424	Br46	Small scale
CH115	CH11	Small scale
Ef53	Ef6	Large scale
G143	G14	Medium scale
Gt473	Gt51	Medium scale
J131	J13	Small scale
M183	Mg18	Large scale
T345	Ts36	Small scale
T361	T39	Small scale
TE301	Te32	Large scale
TE308	Te32	Large scale
Ts219	Ts23	Medium scale
Ty76	Ty8	Medium scale
Ty83	Ty8	Medium scale

**TABLE 4:** Phenotypic resistance profiles and genotypic antimicrobial resistance determinants of 20 sequenced *E. coli* isolates.

Isolate	Phenotypic antimicrobial resistance profile	Genetic resistance determinants
A296	Ampicillin, ciprofloxacin, azithromycin, nalidixic acid, trimethoprim, sulfamethoxazole	*sul1*, *aadA2*,*mphA*, *bla*_*TEM*_, *dfrA12*, *gyrA*S83L, *gyrA*D87N, *parC*S80I, *parE*S458A
AB04	Ampicillin, ciprofloxacin, nalidixic acid, trimethoprim, sulfamethoxazole	*tetA*, *dfrA1*, *aadA1*, *sul1*, *aph(3′)-lb*, *mph(B*), *aph(6)-ld*, *bla*_*TEM*_, *sul2*, *gyrA*S83L, *gyrA*D87Y, *gyrA*D678E, *parC*S80I
Ah232	Ciprofloxacin, azithromycin, chloramphenicol, nalidixic acid, tetracycline, trimethoprim	*tetA*, *mphA*, *catA1*, *dfrA14*, *gyrA*S83L, *gyrA*D87H, *gyrA*D678E, *gyrA*D741E, *parC*S80I
Am237	Ciprofloxacin, tigecycline, nalidixic acid, tetracycline, sulfamethoxazole	*tetA*, *sul2*, *aph(3′)-lb*, *aph(6)-ld*, *gyrA*S83L, *gyrA*D87N, *parC*80I, *parE*S458A
Am240	Ciprofloxacin, azithromycin, chloramphenicol, nalidixic acid, tetracycline, trimethoprim	*tetA*,*dfrA14*, *catA1*, *mphA*, *gyrA*S83L, *gyrA*D87H, *gyrA*D678E, *gyrA*D741E, *parC*S80I
B33	Ampicillin, ciprofloxacin, gentamicin, nalidixic acid, tetracycline	*tetA*, *aaC(3)-lld*, *aph(3’)-la*, *bla*_*TEM*_, *gyrA*S83L, *gyrA*D87N, *parC*S80I, *parC*E84G
B424	Ampicillin, ciprofloxacin, tetracycline, trimethoprim, sulfamethoxazole	*2 x tetA*, *aph(3”)-lb*, 2 x *bla*_*TEM*_, *aph(6)-ld*, *dfrA14*, q*nrS*, *sul2*
CH115	Phenotypically susceptible	No acquired resistance gene or mutation
Ef53	Ampicillin, ciprofloxacin, chloramphenicol, nalidixic acid, tetracycline, trimethoprim, sulfamethoxazole	*catA1*, *sul2*, *dfrA36*, *aph(3”)-lb*, *aph(6)-ld*, *tetA*, *bla*_*TEM*_, *gyrA*S83L, *gyrA*D87N, *gyrA*A252G, *parC*S80I, *parC*475E
G143	Ampicillin, ciprofloxacin, chloramphenicol, nalidixic acid, tetracycline, trimethoprim, sulfamethoxazole	*tetA*, *dfrA36*, *sul2*, *catA1*, *aph(3”)-lb*, *aph(6)-ld*, *bla*_*TEM*_, *gyrA*S83L, *gyrA*D87N, *gyrA*A252G, *parC*S80I, *parC*475E
Gt473	Ciprofloxacin, nalidixic acid, tetracycline, trimethoprim, sulfamethoxazole	*tetA*, *aadA5*, *sul2*, *dfrA17*, *gyrA*S83L, *gyrA*D87N, *parC*S80I
J131	Ciprofloxacin, tigecycline, nalidixic acid, tetracycline	*tetA*, *gyrA*S83L, *gyrA*D87N, *parC*S80I
M183	Phenotypically susceptible	No acquired resistance gene or mutation
T345	Ampicillin, ciprofloxacin, gentamicin, nalidixic acid, tetracycline	*aph(3’)-la*, t*etA*, *bla*_*TEM*_, *aac(3)-lld*, *gyrA*S83L, *gyrA*D87N, *parC*S80I, *parC*E84G
T361	Ampicillin, ciprofloxacin, tetracycline, trimethoprim, sulfamethoxazole	*bla*_*TEM*_, *aph(3”)-lb*, t*etA*, *aph(6)-ld*, *sul2*, *dfrA14*, *qnrS*
TE301	Ampicillin, ciprofloxacin, azithromycin, nalidixic acid, trimethoprim, sulfamethoxazole	*dfrA12*, *aadA2*, *sul1*, *mphA*, *bla*_*TEM*_, *gyrA*S83L, *gyrA*D87N, *parC*S80I, *parE*S458A
TE308	Ampicillin, ciprofloxacin, azithromycin, nalidixic acid, tetracycline, trimethoprim, sulfamethoxazole	*sul1*, *aadA2*, *dfrA12*, *bla*_*TEM*_, *mphA*, *gyrA*S83L, *gyrA*D87N, *parC*S80I, *parE*S458A
Ts219	Ciprofloxacin, azithromycin, chloramphenicol, nalidixic acid, tetracycline, trimethoprim	*tetA*, *mphA*, *catA1*, *dfrA14*, *gyrA*S83L, *gyrA*D87H, *gyrA*D678E, *gyrA*D741E
Ty76	Ampicillin, ciprofloxacin, nalidixic acid, tetracycline, trimethoprim, sulfamethoxazole	*tetA*, *aph(”)-lb*, *bla*_*TEM*_, *dfrA7*, *aph(6)-ld*, *sul2*, *gyrA*S83L, *gyrA*D87N, *parC*S80I, *parC*L440R, *parE*S458T
Ty83	Ampicillin, ciprofloxacin, amikacin, chloramphenicol, nalidixic acid, tetracycline, trimethoprim, sulfamethoxazole	*cmlA*, *aph(3”)-lb*, *aph(6)-ld*, *tetA*, *dfrA12*, *sul3*, *aadA1*, *aadA2*, *bla*_*TEM*_, *gyrA*S83L, *gyrA*D87N, *parC*S80I

**TABLE 5:** Plasmid-mediated antimicrobial resistance (AMR) and avian pathogenic *Escherichia coli* (APEC)-associated virulence gene carriage in 20 sequenced *E. coli* isolates, showing their predicted plasmid replicon and mobility types, and the related contig number.

Isolate	Predicted plasmid replicon types containing AMR genes	Predicted plasmid replicon types containing virulence genes
A296	p0111(contig5)^b^: *sul1*, *aadA2*, *mphA*, *bla*_*TEM*_, *dfrA12*	IncFI/FII(contig6)^c^: *iss*, *iroN*, *ompT*, *cvaC, hlyF*
AB04	IncFI/FII/Q1(contig11)^a^ *: tetA*, *dfrA1*, *aadA1*, *sul1*, *aph(3)-lb*, *mphB*, *aph(6)-ld*, *bla*_*TEM*_, *sul2*	IncFI/FII/Q1(contig11)^a^: *iss*, *iroN*, *ompT*, *cvaC*, *hlyF*
Ah232	IncFI/FII(contig18)^c^: *tetA*, *mphA*, *catA1*, *dfrA14*	IncFI/FII(contig18)^c^: *ompT, hlyF*, *papC*
Am237	IncY(contig4)^c^: *tetA*, *sul2*, *aph(3)-lb*, *aph(6)-ld*	IncFI/FII(contig15)^c^: *ompT*, c*vaC*, *hlyF*
Am240	IncFI/FII(contig18)^c^: *tetA*, *mphA*, *catA1*, *dfrA14*	IncFI/FII(contig18)^c^: *ompT*, *hlyF*, *papC*
B33	IncFI/FII(contig6)^c^: *aac(3)-IId*, *bla*_*TEM*_ IncX1(contig5)^a^: *aph(3’)-Ia*, *tetA*	IncFI/FII(contig6)^c^: *iss*, *iroN*, *ompT*, *iutA*, *cvaC*, *hlyF*
B424	p0111(contig2)^b^: *bla*_*TEM*_, *tetA*, *qnrS* IncN(contig3)^c^: *tetA, aph(3”)-lb*, *bla*_*TEM*_, *aph(6)-ld*, *dfrA14*, *sul2*	No plasmid-mediated APEC-related virulence genes detected
CH115	No plasmid-mediated AMR genes detected	No plasmid-mediated APEC-related virulence genes detected
Ef53	IncX1(contig33)^c^: *catA1*, *aph(3”)-lb*, *aph(6)-ld*, *tetA*, *bla*_*TEM*_	IncFI(contig2)^c^: *iutA*
G143	IncX1(contig14)^c^: *catA1*, *aph(3”)-lb*, *aph(6)-ld*, *tetA*, *bla*_*TEM*_	IncFIB(contig20)^a^: *hlyF* and *papC*
Gt473	IncY(contig2)^a^: *tetA*, *aadA5*, *sul2*, *dfrA17*	No plasmid-mediated APEC-related virulence genes detected
J131	IncFI/FII(contig5)^c^: *tetA*	IncFI/FII(contig5)^c^: *iss*, *iroN*, *ompT*, *iutA*, *cvaC*, *hlyF*
M183	No plasmid-mediated AMR genes detected	No plasmid-mediated APEC-related virulence genes detected
T345	IncX1(contig3)^a^: *aph(3’)-la*, *tetA* IncFI/FII(contig4)^c^: blaTEM, aac(3)-lld	IncFI/FII(contig4)^c^: *iss*, *iroN*, *ompT*, *iutA*, *cvaC*, *hlyF*
T361	IncFI/FII(contig2)^c^: *bla*_*TEM*_, *aph(3”)-lb, tetA*, *aph(6)-ld*, *sul2*, *dfrA14*, *qnrS*	IncFI/FII(contig2)^c^: *iss*, *tsh*, *iroN*, *ompT*, *iutA*, *cvaC*, *hlyF*
TE301	p0111(contig8)^b^: *sul1*, *aadA2*, *mphA*, *bla*_*TEM*_, *dfrA12*	IncFI/FII(contig17)^c^: *iss*, *iroN*, *ompT*, *cvaC*, *hlyF*
TE308	p0111(contig6)^b^: *sul1*, *aadA2*, *mphA*, *bla*_*TEM*_, *dfrA12*	IncFI/FII(contig5)^c^: *iss, iroN*, *cvaC*, *hlyF*
Ts219	IncFI/FII(contig6)^c^: *tetA*, *mphA*, *catA1*, *dfrA14*	IncFI/FII(contig6)^c^: *ompT*, *hlyF*, *papC*
Ty76	IncFI/FII(contig1)^c^: *tetA*	IncFI/FII(contig1)^c^: i*ss*, *iroN*, *ompT*, *iutA*, *cva*C, *hlyF*
Ty83	IncI1-I(contig1)^c^: *aadA1*, *aadA2*, *cmlA*, *dfrA12*, *sul3* IncFI/FII(contig15)^c^: *aph(3”)-lb*, *aph(6)-ld*, *tetA*, *bla*_*TEM*_	IncFI/FII(contig15)^c^: *iss*, *iroN*, *ompT*, *iutA*, *cvaC*, *hlyF*

*^a^*Non-mobilizable.

*^b^*Mobilizable.

*^c^*Conjugative, as predicted by MOB-suite v3.1.9. via the Solu platform (Robertson and Nash, 2018; Saratto et al., 2025).

The isolates showed expected pathogenic variability for poultry ranging from 0/10 to 7/10 APEC-associated virulence genes with several strains carrying up to 6 APEC-associated virulence markers on conjugative plasmids (Figure 2 and Table 5), as previously described (Sarowska et al., 2019). In addition, the isolates harbored a range of virulence-associated genes additional to the 10 selected APEC-associated virulence genes, including those involved in iron acquisition, adhesion, toxin production, capsule formation and serum resistance encoding genes (Supplementary Table 3), reflecting virulence gene profiles commonly described in extraintestinal pathogenic *E. coli* ExPEC (Malberg Tetzschner et al., 2020).

## 4 Discussion

This study highlights a concerning level of AMR in *E. coli* isolated from broiler chickens during starter-phase in Ethiopia. The isolates exhibited extremely high resistance prevalence (>70%) mainly to ciprofloxacin, nalidixic acid and tetracycline, along with very high resistance to trimethoprim (Table 2), according to EFSA categorization. A very high proportion of multidrug-resistant (MDR) isolates (77%) was observed. The observed MDR prevalence in our study is similar to the recently reported (80%) MDR level in older chickens from conventional poultry farms in central Ethiopia (Tilahun and Efa, 2026). Furthermore, evidence from previous studies shows that MDR levels in older or late-production-stage poultry frequently exceed 90%, further highlighting the widespread nature of multidrug resistance in Ethiopian poultry systems (Shecho et al., 2017; Tigabie et al., 2023). In addition, the tetracycline resistance levels in our study (84%) were also comparable to levels reported previously in older chickens (77.8%) by the same authors (Tilahun and Efa, 2026). The early production phase remains particularly critical because chicks do not have a stable intestinal microbiota yet and are therefore more susceptible to colonization by resistant strains. Once colonization occurs, these resistant bacteria interact with other members of the developing intestinal microbiota, a process that accelerates the ecological and evolutionary spread of AMR through mechanisms such as horizontal gene transfer and selection pressures (Bottery et al., 2021). Our findings contribute new insights by focusing specifically on broilers chickens during the starter phase and by using an extensive antimicrobial panel and robust MIC-based interpretations.

The *E. coli* isolates obtained from broiler chickens during starter phase exhibited considerable genetic heterogeneity (Figure 2). This finding agrees with previous studies (Ajibola et al., 2025; Habib et al., 2025; Tang et al., 2025). For some isolates, like for example Am237/Am240 and TE301/TE308, high genetic similarity does not come as a surprise as these strains were collected from the same farms. However, nearly identical isolates such as TE301/TE308 and A296, or Am237/Am240, Ah232, and Ts219 were also recovered from different farms. Such evidence may reflect shared contamination pathways or the ongoing circulation of genetically similar *E. coli* strains among farms, and might also indicate strain movement via chicks originating from the few available day-old chick suppliers. Complementing these findings, fifteen different serotypes and considerable (cg)MLST diversity were observed among the sequenced isolates in the current study (Figure 2), confirming heterogeneous AFEC populations in apparently healthy broiler chicks. Among the detected serotypes, O21:H16 and O8:H23 were the most common in the current isolates, while MLST identified ST224 and ST93 as the predominant sequence types. O21:H16 has been reported among broiler *E. coli*, showing close phylogenetic relatedness to human and broiler ExPEC, suggesting potential zoonotic links at the population level (Yang et al., 2021). Similarly, the detection of ST224 and ST93 indicates the circulation of genetically related strains carrying virulence-associated determinants previously recorded among poultry lineages (da Silva et al., 2022; Maluta et al., 2014). Notably, the specific serotype O8:H23 is rarely documented in poultry; nevertheless, the O8 serogroup was identified among APEC isolates in a broader poultry study (Runcharoon et al., 2025). Overall, the observed strain distribution may align with genomic surveys highlighting extensive serotype/ST heterogeneity in broiler *E. coli*, underscoring that strains with pathogenic potential are not confined to a single lineage but arise across diverse genetic backgrounds.

The highest prevalence of acquired resistance across all antimicrobial classes were observed for the fluoroquinolones (Table 2). Although the agreement between epidemiological (ECOFF) and clinical (breakpoint) interpretive criteria is less pronounced for this class than for other classes, the consistently high resistance prevalence remain deeply concerning. As fluoroquinolones are classified as highest-priority critically important antimicrobials reserved for last-resort treatment, these findings underscore a serious threat to their therapeutic value. Compounding this issue, ECOFF-based resistance reporting in Ethiopian poultry farms is uncommon, which limit the completeness of national AMR surveillance and hindering efforts to assess the true magnitude of the problem. When compared with previous studies from other regions, the resistance levels observed in this study were higher than those reported in Tanzania (Maganga et al., 2023), but lower than the findings from Bangladesh (Ibrahim et al., 2023b). Additionally, factors such as sample characteristics and local environmental conditions may also contribute to the prevalence observed (Szoke et al., 2025).

Furthermore, the resistance to tetracycline in the current study was 84%, which is higher than previously reported as ranging from 46.3% to 75% in Ethiopia (Bushen et al., 2021; Hailu et al., 2024; Messele et al., 2017; Sarba et al., 2019), as well as higher than 67.8% reported in Tanzania (Maganga et al., 2023) and 53% in Uganda (Mbatidde et al., 2024). The high level of tetracycline resistance observed in broiler production is likely linked to the frequent use of this antimicrobial agent in veterinary practice (Etefa et al., 2021; Gemeda et al., 2020; Beyene et al., 2016). Moreover, some AFEC strains may also carry resistance determinants before chicks arrive on the farm, suggesting that early-life microbial reservoirs could play a role in the development and persistence of AMR. Even though day-old chicks were not sampled in current investigation, they have been suggested to be an important source of antimicrobial resistant *E. coli* for poultry flocks before (Nedbalcova et al., 2023; Ringenier et al., 2026). Other findings (Austin et al., 1999) demonstrated that prolonged antimicrobial pressure is required before measurable resistance can emerge. This highlights the importance of surveillance systems that incorporate both historical and current data and resistance data in order to more accurately capture the temporal dynamics of resistance.

In the current study, a high level of trimethoprim resistance (53%) was detected among *E. coli* isolates from broiler chickens. According to (European Food Safety Authority [EFSA], 2025) reporting categories, this falls within the very high resistance range. However, it was lower than findings reported elsewhere in the world, such as in Nigeria and Brazil, where resistance exceeded 80% (Al-Mustapha et al., 2022; Dos Anjos Adur et al., 2022). In contrast, our result is quite nearly consistent with findings from Portugal (Manageiro et al., 2017). Such variation may be explained due to factors like the concurrent administration of trimethoprim with other antimicrobials in poultry production, which can influence selective pressure and resistance patterns.

Multiple AMR determinants were identified using different resistance gene databases. Even though the Reference Gene Catalog and the Card Database generated the most extensive list of putative AMR-associated genes and mutations, the ResFinder database appeared more focused on acquired resistance determinants with a clear phenotypic relevance. For example, both the Reference Gene Catalog and the Card Database indicated the presence of the *blaEC* gene, even though this gene is intrinsically present in most *E. coli* strains and only results in acquired AMR when point mutations are present in its promoter region (Totté et al., 2025). In addition, several genes were detected with low sequence identity or substantial mismatches. For example, all sequenced strains carried a *tet34* variant showing only 68% identity and nearly 50 mismatches compared to the reference gene. Similar *tet34* variants have recently been described in *E. coli* and *Vibrio* isolates (Abdalla et al., 2025; Sebastian et al., 2025), but their functional relevance remains uncertain. Likewise, other AMR genes [e.g., *aph(3’)-Ia, aph(3”)-lb, blaTEM, sul2*] were detected with low match strength (Supplementary Table 4), and were therefore excluded from further analysis, as their contribution to phenotypically detectable resistance is unlikely. Functional relevance was also considered for macrolide resistance genes. In contrast to *mphA*, which is associated with azithromycin resistance, *mphB* has recently been shown not to confer acquired macrolide resistance in Enterobacterales (Ivanova et al., 2024) and its presence was therefore not reported. Enterobacterales are intrinsically resistant to most macrolides, including azithromycin. Therefore, from a (veterinary) clinical point of view, there seems little added value of looking into acquired resistance against azithromycin. However, the aim of current research was to evaluate whether avian fecal *E. coli* act as important reservoirs of antimicrobial resistance genes. As stated by CLSI, azithromycin can be used to treat *Salmonella* (typhoid fever) or *Shigella* infections, and azithromycin clinical breakpoints for *Shigella* and *Salmonella* have been established. In Enterobacterales, acquired resistance against azithromycin can be mediated by few genes, including *mphA* (Ayele et al., 2025; Debnath et al., 2025; Ma et al., 2024). Current data therefore show that AFEC strains can be reservoirs for *mphA* genes and therefore may contribute to the persistence or dissemination of azithromycin resistance genes to other, possibly clinically relevant, Enterobacterales, like *Salmonella* or *Shigella*.

Several AMR-related mutations were identified. Mutations in *gyrA*, *parC* and *parE* were considered phenotypically relevant, as their presence likely explains the increased fluoroquinolone MIC values observed (Figure 2). Conversely, mutations in *cyaA*, *gltP*, and *uhpT* (associated with fosfomycine resistance) and in *nfsA/B* (associated with nitrofurantoin resistance), could not be phenotypically evaluated, as these antimicrobials were not included in the currently used MIC panel. Moreover, recent evidence indicates that at least some of these mutations may be functionally silent (Dulyayangkul et al., 2024). Overall, these findings highlight that genomic AMR detection tools may overestimate acquired resistance potential if intrinsic resistance genes, low-identity matches, or non-functional mutations are not carefully interpreted. Integration with phenotypic susceptibility testing remains essential to avoid over- or misinterpretation of genomic AMR data (Feldgarden et al., 2019; Matsumura et al., 2025; Skoulakis et al., 2025).

The concordance between phenotypic resistance and genotypic findings strengthens the reliability of current results. Phenotypic resistance was consistently supported by the presence of corresponding resistance genes and mutations for all antimicrobials for which both genotypic and phenotypic results were available. The results for highest priority, critically important antimicrobial agents present a nuanced picture, revealing two distinct and opposing outcomes. The high prevalence of fluoroquinolone resistance is extremely worrying, while the absence of resistance to cephalosporins, colistin, and meropenem is encouraging.

The detection of multiple APEC-associated virulence gene in cloacal *E. coli* isolates is consistent with the view that AFEC populations may serve as reservoirs for extra intestinal pathogenic lineages, such as APEC, as suggested previously (Song et al., 2025). The high detection level of conjugative plasmids carrying multiple resistance and/or virulence genes underscores the risk of horizontal gene transfer within and between poultry flocks. IncF and IncX plasmids were particularly prevalent, consistent with previous studies in poultry and human clinical isolates (Juraschek et al., 2021; Rozwandowicz et al., 2018). Indeed, some of the currently observed plasmids show characteristics typically described for ColV virulence plasmids. These large, conjugative plasmids of IncF-type with genes encoding iron acquisition systems, serum resistance, colicin production and other virulence traits in a genetic backbone with high similarity to published ColV plasmids therefore probably represent sublineages of ColV virulence plasmids (Supplementary Figure 1). The co-location of resistance and virulence genes in the multiple isolates and even on the same plasmids raises additional concerns about the emergence of highly virulent and resistant strains.

### 4.1 Limitations of the study

This study has limitations, namely the number of sequenced isolates was relatively small limiting the generalizability of the findings. Sequencing all isolates was not feasible as a result of resource limitations. Second, the study focused exclusively on broilers, thereby excluding layer flocks which operate under distinct production systems and longer lifespans, thus narrowing the scope of inference to a single production type. Taken together, these limitations highlight the need for caution when extrapolating the results to broader poultry populations.

## 5 Conclusion

This study demonstrates the value of integrating phenotypic and genomic data for AMR surveillance. To the best of our knowledge, it provides the first insights into AMR and virulence-associated genes in *E. coli* from broiler chickens during the starter phase in commercial farms in Ethiopia using both phenotypic characterization and WGS. Moreover, a high proportion of MDR *E. coli* isolates was observed, with strains exhibiting diverse resistance profiles and carrying virulence associated genes. The study also revealed resistance to several commonly used antimicrobial agents, including fluoroquinolones. In addition, the high detection level of conjugative plasmids carrying multiple resistance and/or virulence genes underscores the risk of horizontal gene transfer within and between poultry flocks. Virulence genes were frequently detected on key plasmid types, particularly IncF and IncX. To address these concerns, strengthening antimicrobial stewardship, improving farm-level biosecurity, and enhancing diagnostic capacity are essential steps toward mitigating AMR in broiler production systems in Ethiopia, along with further research into early life sources of AMR, including the potential role of day-old chicks. Furthermore, given the high AMR prevalence observed, an urgent need to implement an integrated AMU–AMR surveillance program in Ethiopia’s poultry production systems.

## Data Availability

The datasets presented in this study can be found in online repositories. The names of the repository/repositories and accession number(s) can be found in the article/Supplementary material.

## Footnotes

1
https://mic.eucast.org/search/

2
https://cge.food.dtu.dk/services/MLST/

3
https://sarredondo.shinyapps.io/mlplasmids
