# Supplementary material for: Phenotypic and genomic analysis of antimicrobial resistant Escherichia coli isolates obtained from starter-phase broilers of commercial chicken farms in central Ethiopia
Source: Front Microbiol. 2026 Jun 19;17:1848618. doi: 10.3389/fmicb.2026.1848618 (PMC13328261; doi:10.3389/fmicb.2026.1848618)
Supplement: Supplementary file 1 [file Data_Sheet_1.zip › Supplemental files/Supplemental File Table 2.docx]

Supplementary Table 2: The proportion of *E. coli* isolates recovered from samples analyzed according to farm size

| **Farms** | **Scale of Farms** | **Number of samples tested** | **Number of *E. coli* positives** | **(%) *E. coli* isolated** |
| --- | --- | --- | --- | --- |
|  | **Large scale** |  |  |  |
| AB1 |  | 10 | 6 | 60 |
| E5 |  | 11 | 5 | 45.45 |
| Ef6 |  | 11 | 6 | 54.55 |
| Mg18 |  | 10 | 5 | 50 |
| J20 |  | 10 | 5 | 50 |
| Mt27 |  | 11 | 3 | 27.27 |
| Hy28 |  | 10 | 5 | 50 |
| My29 |  | 10 | 6 | 60 |
| Te32 |  | 10 | 4 | 60 |
| Wb40 |  | 10 | 4 | 40 |
| K41 |  | 10 | 5 | 50 |
| Zd43 |  | 10 | 4 | 40 |
|  | **Subtotal** | **123** | **58** | **47.97** |
|  | **Medium scale** |  |  |  |
| M7 |  | 9 | 6 | 66.67 |
| Ty10 |  | 10 | 6 | 66.67 |
| G14 |  | 9 | 5 | 55.56 |
| Z16 |  | 9 | 6 | 66.67 |
| D17 |  | 9 | 3 | 33.33 |
| H19 |  | 9 | 5 | 55.56 |
| N21 |  | 9 | 6 | 66.67 |
| F22 |  | 9 | 4 | 44.44 |
| Ts23 |  | 10 | 6 | 60 |
| Ah24 |  | 9 | 5 | 55.56 |
| Am25 |  | 9 | 5 | 55.56 |
| Nt26 |  | 9 | 6 | 66.67 |
| Tm30 |  | 9 | 4 | 44.44 |
| Al31 |  | 9 | 5 | 55.56 |
| Ey33 |  | 9 | 6 | 66.67 |
| Nl34 |  | 9 | 6 | 66.67 |
| Sf35 |  | 9 | 3 | 33.33 |
| Jt44 |  | 9 | 4 | 44.44 |
| Ge45 |  | 9 | 5 | 55.56 |
| Bk48 |  | 9 | 5 | 55.56 |
| Ad50 |  | 9 | 4 | 44.44 |
| Gt51 |  | 9 | 5 | 55.56 |
| Ng52 |  | 9 | 4 | 44.44 |
|  | **Subtotal** | **209** | **114** | **54.55** |
|  | **Small scale** |  |  |  |
| S2 |  | 9 | 6 | 66.67 |
| T3 |  | 9 | 4 | 44.44 |
| B4 |  | 9 | 5 | 55.56 |
| Mk9 |  | 9 | 4 | 44.44 |
| W10 |  | 9 | 5 | 55.56 |
| CH11 |  | 9 | 6 | 66.67 |
| IB12 |  | 9 | 4 | 44.44 |
| J13 |  | 9 | 5 | 55.56 |
| Mf15 |  | 9 | 5 | 55.56 |
| Ts36 |  | 9 | 6 | 55.56 |
| Yo37 |  | 9 | 5 | 55.56 |
| R38 |  | 9 | 4 | 44.44 |
| T39 |  | 9 | 6 | 66.67 |
| Ay42 |  | 9 | 4 | 44.44 |
| Br46 |  | 9 | 6 | 66.67 |
| Kb47 |  | 9 | 4 | 44.44 |
| Db49 |  | 9 | 5 | 55.56 |
|  | **Subtotal** | **153** | **84** | **54.90** |
|  | **Grand Total** | **485** | **256** | **52.99** |

Supplementary Table 2: Overall proportion of E. coli yielding across the three production scales

| Farm scale | Number of samples tested | Number of *E. coli* positives | Prevalence (%) | Chi square value (P value) |
| --- | --- | --- | --- | --- |
| Large scale | 123 | 58 | 47.97 | 1.67 (0.43) |
| Medium scale | 209 | 114 | 54.55 |  |
| Small scale | 153 | 84 | 54.90 |  |
| Total | **485** | **256** | **52.99** |  |
