# Supplementary figures and images for: Phenotypic and genomic analysis of antimicrobial resistant Escherichia coli isolates obtained from starter-phase broilers of commercial chicken farms in central Ethiopia
Source: Front Microbiol. 2026 Jun 19;17:1848618. doi: 10.3389/fmicb.2026.1848618 (PMC13328261; doi:10.3389/fmicb.2026.1848618)

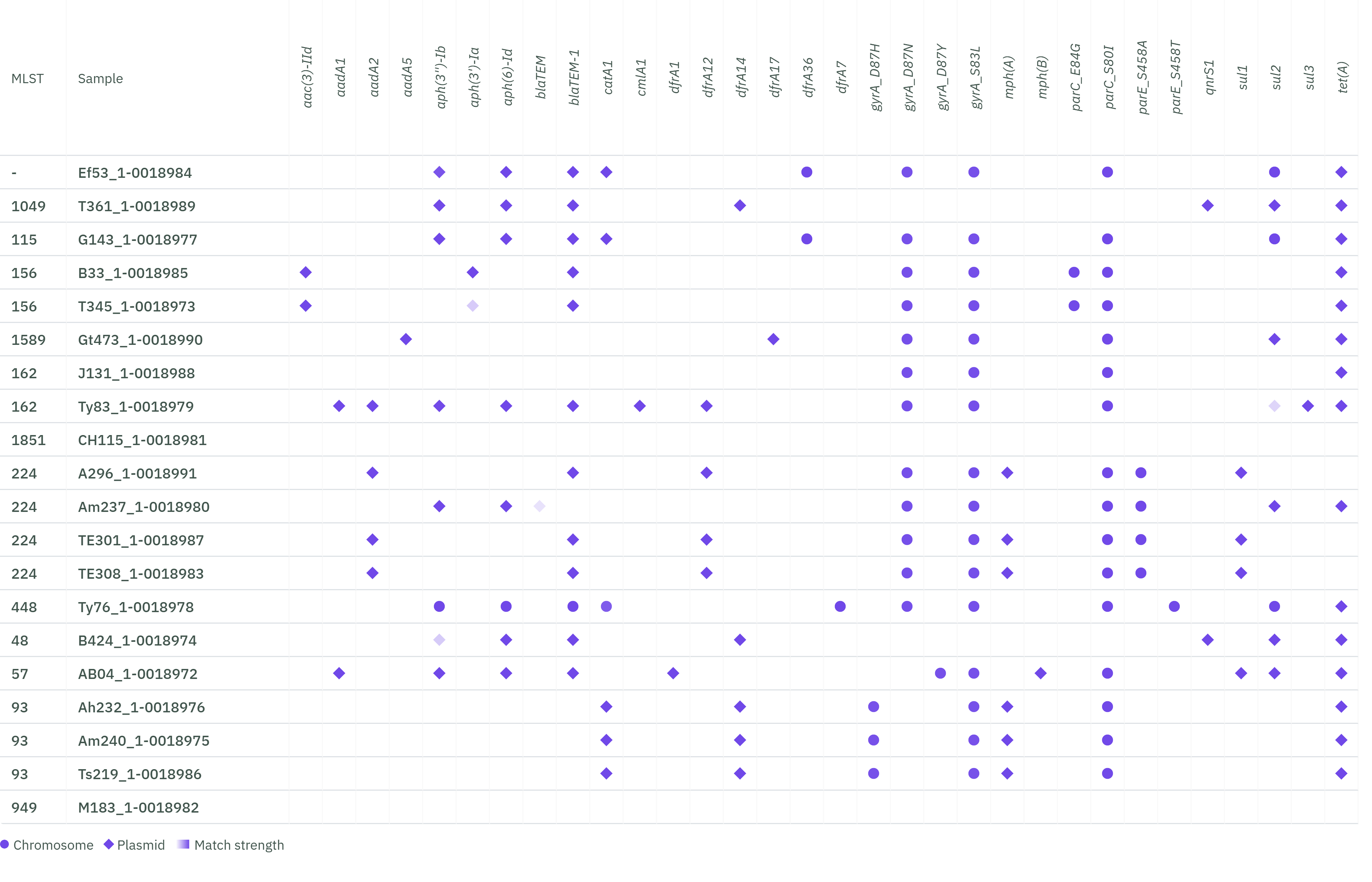

Supplement: Supplementary file 1 [file Data_Sheet_1.zip › Supplemental files/Supplemental File Table 4.png]

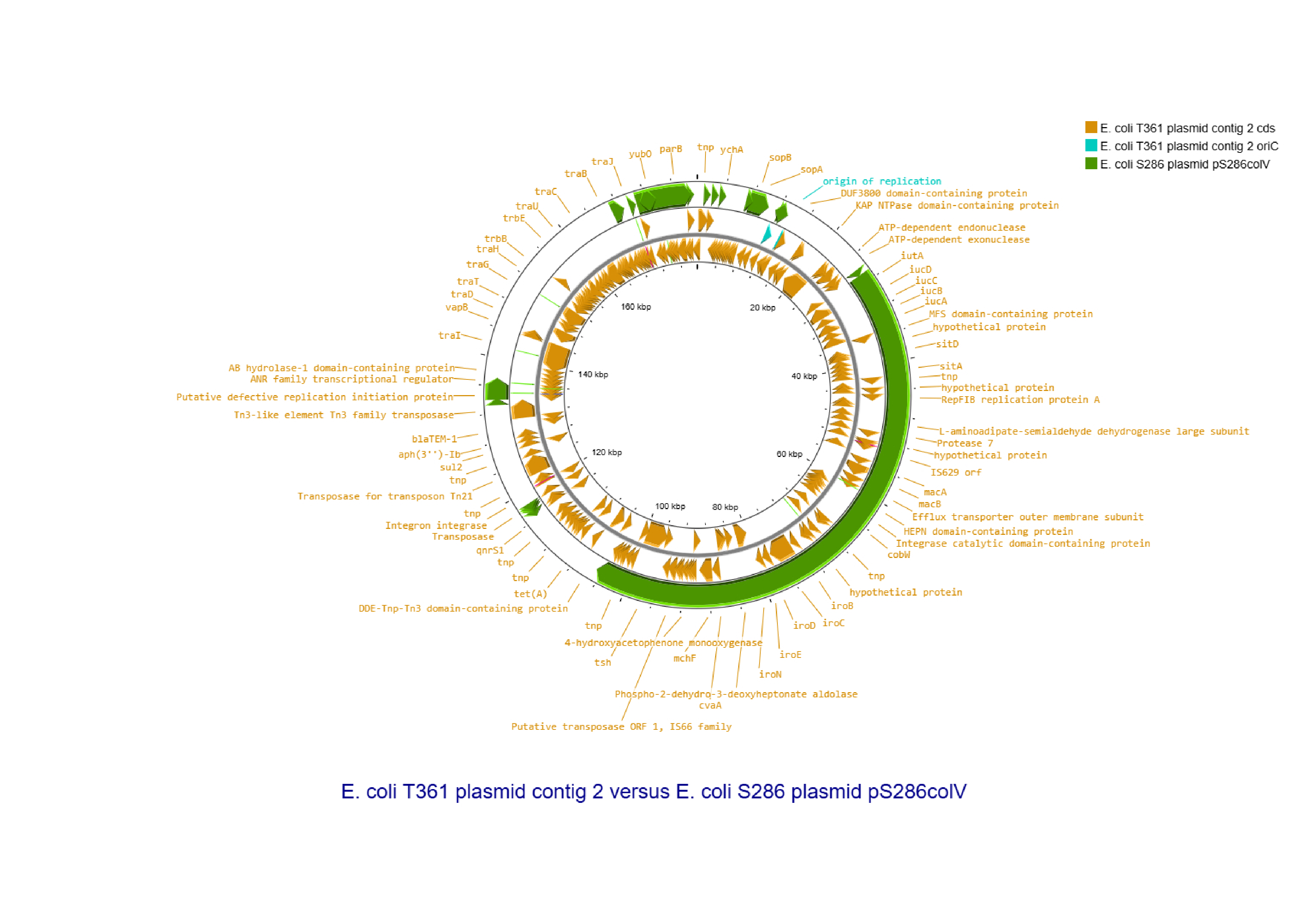

Supplement: Supplementary file 2 [file Image_1.tiff]
